# Embolic Signals during Routine Transcranial Doppler Ultrasonography in Aneurysmal Subarachnoid Hemorrhage

**DOI:** 10.1155/2015/153714

**Published:** 2015-03-29

**Authors:** Fernando Mendes Paschoal, Karla de Almeida Lins Ronconi, Marcelo de Lima Oliveira, Ricardo de Carvalho Nogueira, Eric Homero Albuquerque Paschoal, Manoel Jacobsen Teixeira, Eberval Gadelha Figueiredo, Edson Bor-Seng-Shu

**Affiliations:** Laboratory for Neurosonology and Cerebral Hemodynamics, Division of Neurological Surgery, Hospital das Clinicas, Sao Paulo University Medical School, 04040-001 Sao Paulo, SP, Brazil

## Abstract

*Introduction*. Cerebral emboli may occur in subarachnoid hemorrhage (SAH) and intracranial aneurysm surgery. Although embolic signs (ES) have been reported in SAH, their origin remains unclear. The aim of this study was to report the detection of ES during routine TCD monitoring in patients with aneurysmal SAH. *Methods*. A total of 105 patients with aneurysmal SAH were submitted to TCD evaluation. Patients were monitored almost daily (5 times per week). In each monitoring session, one experienced operator performed TCD to detect or assess vasospasm and ES in arteries of the Willis polygon. *Results.* Four patients out of a total of 105 patients with aneurysmal SAH were found to present spontaneous cerebral embolization during routine TCD monitoring. The average age of the 4 patients (mean ± standard deviation) was 59.5 ± 8.34 years (range 49–68 ys); female patients predominated representing 75% (3/4) of subjects. *Conclusion*. Although detection of ES was relatively rare in this study, rates of emboli occurrence may be higher under systematic monitoring. The detection of ES after SAH surgery reinforces the need to study the role of embolus in this condition and may be an indicator for prophylactic antithrombotic treatment.

## 1. Introduction

Cerebral vasospasm is considered a common and serious complication of aneurysmal subarachnoid hemorrhage (SAH), contributing to elevated rates of morbidity and mortality. Cerebral ischemia due to vasospasm has traditionally been thought to result from reductions in cerebral blood flow through constricted vessels [1, 2]. Recently, cerebral ischemia associated with vasospasm is considered as a result of complex interactions among cerebral blood flow, metabolism and inflammation [3].

Some authors have observed thrombi in aneurysmal sac and vessels in which vasospasm had resulted in cerebral ischemia [4, 5]. Thrombus in the aneurysmal sac may result from turbulence and slow blood flow and can act as a source of distal embolization. However their origin remains unclear as does their contribution to brain ischemia [1–5].

Transcranial Doppler (TCD) ultrasonography is used routinely in some centers to monitor SAH patients. This technique can detect both cerebral vasospasm and embolization. Recently, cerebral embolization has been described during TCD monitoring in SAH setting. The aim of this study was to report the detection of ES during routine TCD monitoring in patients with aneurysmal SAH.

## 2. Methods

A total of 105 patients with aneurysmal SAH admitted to the Hospital das Clínicas of University of São Paulo Medical School, Brazil, between 2009 and 2010, were investigated in a prospective design. This study was approved by the local research ethics committee. The average age of the patients (mean ± standard deviation) was 51.52 ± 13.03 years (range 23–87 ys); female patients predominated representing 57% (60/105) of subjects. Among the patients, 35.2% (37/105) were grade I on the Hunt-Hess clinical scale while 51.4% (54/105) were grade IV on the Fisher CT scale. Based on angiographic findings, the most common sites of aneurysms were the anterior communicating artery (AnCoA) (36/105), posterior communicating artery (PCoA) (33/105), middle cerebral artery (31/105), and others (24/105). Exclusion criteria were atrial fibrillation, recent myocardial and cerebral infarction, valvular heart disease, ulcerated carotid and vertebral atherosclerotic plaque, and nonaneurysmal SAH.

Demographic, clinical, and radiological variables including age and sex, date of SAH, primary neurological deficit, angiographic findings, surgical management, and CT scan findings were recorded for each patient.

The Fisher scale was used for grading CT scan findings while the Hunt-Hess score was employed for clinical severity [1]. Focal neurological deficits due to vasospasm were assessed by thorough neurological examination. Symptomatic vasospasm was defined as a focal neurological deficit not due to rebleeding, hydrocephalus, metabolic abnormalities, or surgical and angiographic complications. Cerebral angiography was performed for diagnosing aneurysms in all patients. However, angiographic criteria were not used for determining vasospasm.

TCD evaluation was performed using an ultrasonographic device (EME Companion/Nicolet) equipped with a 2 MHz probe. Patients were monitored almost daily during the first 2 weeks of their inpatient stay. One experienced operator performed TCD without using specific software for emboli monitoring. Cerebral embolization was suspected during the examination and, afterwards, reviewed off-line. TCD-vasospasm was defined and graded according to previous studies [6, 7]. ES were defined as hyperintensity signals that were random, unidirectional, and of short duration and were producing a characteristic chirping sound [8]. All patients underwent TCD examination before and after surgical or endovascular aneurysm treatment, and the arteries of the carotid system and vertebrobasilar system were evaluated separately, each artery by TCD.

Statistical analysis was used to evaluate the relationship among the presence of ES, vasospasms, and other demographic factors.

## 3. Results

Four out of 105 patients with aneurysmal SAH were found to present spontaneous cerebral embolization during routine TCD monitoring (Table 1). The average age of the patients (mean ± standard deviation) was 59.5 ± 8.34 years (range 49–68 years); female patients represented 75% (3/4) of subjects. Among the selected patients, 50% (2/4) were grade II on the Hunt-Hess clinical scale while 75% (3/4) were grade III on the Fisher CT scale. Based on angiographic findings, the most common sites of aneurysms were the anterior communicating artery (AnCoA) (2/4) and posterior communicating artery (PCoA) (2/4), whereas one patient had more than one aneurysm (middle cerebral artery, PCoA, and choroidal artery). Vasospasm was detected in all patients with embolic events (4 patients) beginning, on average, 5 days after SAH (range 4–6 days). In one patient (25%), the spasm was detected bilaterally in anterior circulation and the basilar artery. Symptomatic vasospasm was present in all patients (100%).

**Figure 1:**
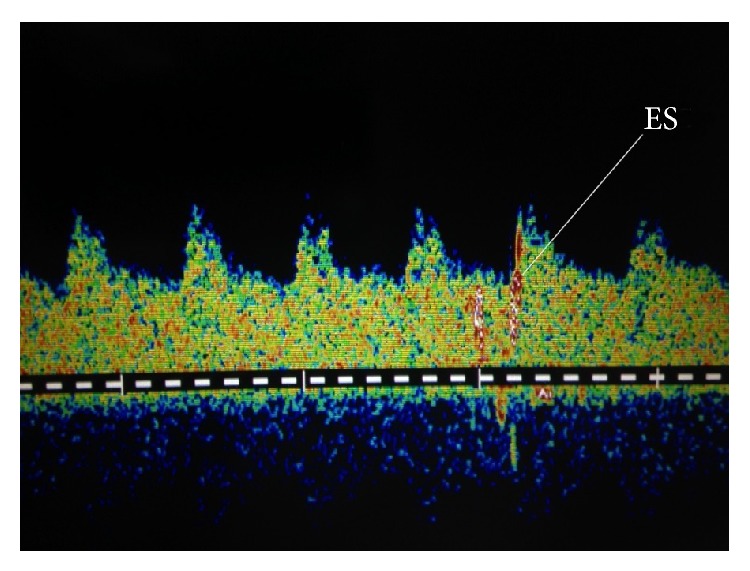
TCD with ES detected in middle cerebral artery.

ES were first detected an average of 7 days after SAH (range 5–9). The ES detection rate was 3.8% (4/105) of patients monitored in the acute phase after SAH (Figure 1). ES were detected in both MCAs and basilar artery for 25% (1/4) of patients, in only MCA for 25% (1/4), in only carotid siphon for 25% (1/4), and in only basilar artery for 25% (1/4).

All patients with cerebral embolic activity underwent early aneurysmal surgical clipping (Table 1).

## 4. Discussion

Cerebral ischemia secondary to vasospasm is an important cause of death and disability following aneurysmal SAH [9]. The pathogenesis of cerebral vasospasm after SAH is not fully understood, but attention has focused on the role of inflammatory responses and immunological reactions to a chemical factor, probably oxyhaemoglobin, among others [4, 10, 11]. In addition, increase in brain metabolic rates due to high glutamate concentration, seizures, and cortical spread depolarization at the time of vasospasm can lead to uncoupling of cerebral flow and metabolism [3].

Cerebral emboli may occur in SAH and intracranial aneurysm surgery. Although ES have been reported in SAH, their origin remains unclear as does their contribution to brain ischemia [12, 13]. The detection of ES provides important pathophysiological information in a variety of disorders, but the clinical importance and possible therapeutic implications of these signals are still under debate [11–15].

Damage to the endothelial wall that occurs associated with aneurysmal SAH may induce microthrombosis and emboli; possible emboli sources include spastic arterial segments, thrombus in an aneurysmal sac, surgical complications, and hypercoagulable states. Giller et al. [11] observed MES in 11 out of 278 (3.95%) patients after aneurysm surgery. In the present study, ES were detected in 4 out of 105 (3.80%) aneurysmal SAH patients during routine monitoring by TCD after aneurysm surgery. Qureshi et al. [10] observed embolization from the aneurysmal sac in 3.3% of the 269 SAH patients, whereas 3% of the 130 patients in the series reported by Wiebers et al., [11] and 6.3% of the 111 patients in the series reported by Raps et al. [14] exhibited ischemic symptoms distal to unruptured aneurysms. It has also been hypothesized that recently coiled or clipped aneurysms may be sources of emboli. In one series of clipped aneurysms, ES were detected in 4% of cases [11], although, akin to the present study, ES were detected during routine vasospasm monitoring without dedicated ES monitoring sessions after clipped aneurysms.

Romano et al. monitored 23 patients with aneurysmal SAH; ES were detected in 70% of patients and one-third of all vessels monitored [15]. In an investigation by Azarpazhooh et al. (2009), ES were detected in 7 out of 27 (26%) patients with aneurysmal SAH [16]. The studies that showed a higher rate of ES used a specific technique for detecting emboli while monitoring lasted more than 30 minutes in each arterial segment studied, contrary to our study which employed routine TCD examination to detect ES.

Patients of our study had concomitantly both cerebral vasospasm and ES. It is possible that spastic arterial segments may have played a role in ES formation [17–19]. Multiple ES were detected in one patient (25%) of our study who had severe vasospasm in bilateral MCA and basilar artery. In the absence of a clear cardiac or carotid artery source for these findings, it is likely that microemboli were generated within the large intracranial vessels [20–22]. Subarachnoid blood products may induce a generalized hyperaggregable condition that is not limited to the spastic artery and results in emboli formation [23, 24].

There were several limitations related to this study, in particular the small sample size. Although detection of emboli was relatively rare in this study, rates of emboli occurrence may be higher under systematic monitoring. The detection of ES after SAH surgery may be an indicator for prophylactic treatment. Future studies may include the description of clot presence in the aneurysm before and during aneurismal surgical treatment.

## Figures and Tables

**Table 1:** Clinical data for 4 patients with subarachnoid hemorrhage (SAH).

Number	Sex	Age	Hunt-Hess score	Fisher grade	Aneurysm location	Surgery	Symptomatic vasospasm	Time/vasospasm and severity	ES detection/site
1	M	64	IV	III	AnCoA	Early clipped aneurysm	Yes	Day 4/diffuse severe	Days 5, 6, and 7/bilateral MCA and basilar artery

2	F	57	II	III	Right MCA/PCoA/ right choroidal	Early clipped aneurysm	Yes	Day 6/right MCA and ACA severe	Day 7 only right carotid siphon

3	F	68	III	IV	AnCoA	Early clipped aneurysm	Yes	Day 4/right MCA moderate left MCA severe	Days 8-9/right MCA

4	F	49	II	III	Left PCoA	Early clipped aneurysm	Yes	Day 6/basilar moderate	Days 9-10/basilar artery

AnCoA = anterior communicating artery; MCA = middle cerebral artery; ACA = anterior cerebral artery; PCoA = posterior communicating artery.
